# Background calcium induced by subthreshold depolarization modifies homosynaptic facilitation at a synapse in *Aplysia*

**DOI:** 10.1038/s41598-019-57362-2

**Published:** 2020-01-17

**Authors:** Bjoern Ch. Ludwar, Klaudiusz R. Weiss, Elizabeth C. Cropper

**Affiliations:** 1 Department of Neuroscience and Friedman Brain Institute, Icahn School of Medicine at Mount Sinai, One Gustave L. Levy Place, New York, NY 10029 USA; 2 Department of Biology and Environmental Sciences, Longwood University, 201 High Street, Farmville, VA 23909 USA

**Keywords:** Synaptic transmission, Short-term potentiation

## Abstract

Some synapses show two forms of short-term plasticity, homosynaptic facilitation, and a plasticity in which the efficacy of transmission is modified by subthreshold changes in the holding potential of the presynaptic neuron. In a previous study we demonstrated a further interactive effect. We showed that depolarizing changes in the presynaptic holding potential can increase the rate at which facilitation occurs. These experiments studied synaptic transmission between an *Aplysia* sensory neuron (B21) and its postsynaptic follower, the motor neuron (B8). We have also shown that subthreshold depolarizations of B21 produce widespread increases in its [Ca^2+^]_i_ via activation of a nifedipine-sensitive current. To determine whether it is this change in ‘background’ calcium that modifies synaptic transmission we compared the facilitation observed at the B21-B8 synapse under control conditions to the facilitation observed in nifedipine. Nifedipine had a depressing effect. Other investigators studying facilitation have focused on Ca_res_ (i.e., the calcium that remains in a neuron after spiking). Our results indicate that facilitation can also be impacted by calcium channels opened before spiking begins.

## Introduction

A well-characterized form of short-term synaptic plasticity is homosynaptic facilitation or short-term enhancement (STE), i.e., repeated presynaptic stimulation induces a progressive increase in postsynaptic potential (PSP) amplitude^1,2^. Additionally, at some synapses the efficacy of spike-mediated synaptic transmission is modified by changes in the holding potential of the presynaptic neuron^3–14^. For example, synaptic transmission is potentiated if spikes are triggered at a more depolarized potential (even when firing frequency is kept constant). Both forms of plasticity can be present at the same synapse^4,15^. This leads to the possibility that they will interact. In a previous study we identified a synapse where interaction occurs^15^. At this synapse holding potential increased the rate at which PSPs facilitated. A question not addressed was, what is the mechanism for this interaction?

In a number of systems subthreshold changes in membrane potential open relatively low voltage activated (LVA) calcium channels^3,8,11,13,14^. This results in global (background) increases in the [Ca^2+^]_i_ that are distinctly different from the spike induced highly localized increases in [Ca^2+^]_i_ that are generally necessary for synchronous transmitter release (Ca_local_). Research in a number of systems has demonstrated that increases in background calcium can have a significant effect on synaptic transmission. For example, it can potentiate it in a graded manner^3,8,11,13,14^. Since changes in holding potential can also increase the rate at which facilitation occurs, a question that we ask in this study is; are effects of holding potential on facilitation at least in part mediated by changes in background calcium? We address this question in experiments conducted in an experimentally advantageous synapse in the mollusc *Aplysia californica*. The presynaptic neuron is an identifed mechanoafferent neuron (B21), which makes a monosynaptic excitatory chemical connection with a motor neuron (B8)^16,17^.

## Materials and Methods

### Preparation

Experiments were conducted on *Aplysia californica* (100–400 g) obtained from Marinus Scientific (Long Beach, CA). *Aplysia* are hermaphrodites, i.e., are both male and female. Animals were anesthetized by injection of an isotonic MgCl_2_ solution that was ~50% of body weight. Buccal ganglia were desheathed and continuously superfused with artificial seawater (ASW) cooled to 14–16 °C as has been described^18^.

### Reagents

Experiments were conducted in ASW with the following composition in mM: 460 NaCl, 10 KCl, 55 MgCl_2_, 11 CaCl_2_, and 10 HEPES buffer, pH 7.6. A stock solution of nifedipine (Sigma-Aldrich, St. Louis, MO) was prepared in DMSO. The final DMSO concentration was <1%.

### Imaging

**Figure 1:**
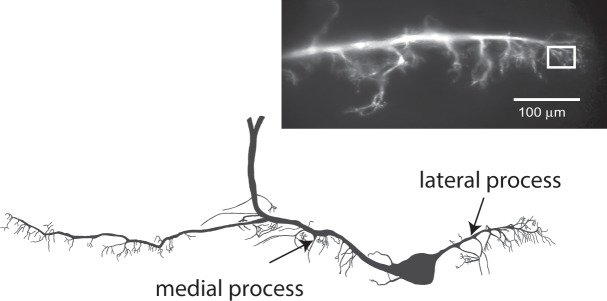
Morphology of B21. A camera lucida drawing of B21. Note that it is a bipolar neuron with major medial and lateral processes. The lateral process is the primary point of contact with the postsynaptic neuron of interest, B8^19^. *Inset*, a lateral process after injection of the Ca^2+^ indicator dye, Calcium Orange. The white box marks a typical ROI.

For most calcium imaging experiments B21 was intracellularly loaded (via iontophoreses) with either Calcium Orange (Thermo Fisher Scientific, Waltham, MA) or Calcium Green 5 N (Thermo Fisher Scientific). Calcium fluorescence was measured as has been described^13,18^. Briefly, we utilized a Nikon FN-1 fixed-stage microscope with a 16×/NA0.8 CFI75 or 10×/0.30w Plan Fluor water immersion lens, and ET-Cy3 or ET-EYFP filter cubes (Chroma Technology, Bellows Falls, VT). Illumination was provided by an X-Cite 120 PC metal halide lamp, and images were acquired with a CoolSNAP HQ^2^ CCD camera (Photometrics, Tucson, AZ) and Nikon NIS Elements AR software (version 3.10). All fluorescence signals were background subtracted and quantified as percent relative change (F − F_0_)/F_0_^18^. B21 is a bipolar neuron with major medial and lateral processes (Fig. 1). The primary point of contact with B8 is the lateral process^19^, consequently imaging was performed in this part of the neuron (e.g., inset in Fig. 1).

To determine whether nifedipine had a significant effect on background calcium we performed ratiometric imaging using the dye Fura-2 (Sigma), which was detected with an ET-FURA 2 filter set (Chroma Technology) and a Ludl motorized filter wheel controlled by MetaFluor version 7.65 imaging software (Molecular Devices, Sunnyvale, CA).

### General electrophysiological techniques

Electrophysiological recordings were obtained using sharp glass electrodes filled with 3 M KAc and 30 mM KCl (typical impedance 7 MΩ). Signals were amplified with an AxoClamp 2B (Molecular Devices) or an SEC-10LX amplifier (x10 headstage, npi electronic, Tamm, Germany), filtered with a model 410 instrumentation amplifier (Brownlee Precision, San Jose, CA) and digitized with a Power 1401 A/D converter (Cambridge Electronic Design (CED), Cambridge, UK) and Spike II software (CED) version 7.01–7.08.

### Protocol used to stimulate B21

**Figure 2:**
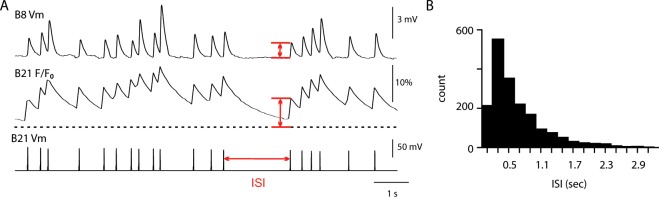
Protocol for experiments in which B21 was stimulated using random interstimulus intervals (ISIs) and changes in [Ca^2+^]_i_ and PSP induction were monitored simultaneously. (**A**) Sample traces from a typical experiment. The bottom trace is a current clamp recording from the soma of B21. The middle trace plots changes in calcium fluorescence quantified as percent relative change (F − F_0_)/F_0_. The top trace is a current clamp recording from B8. In all three traces the red arrows indicate how measurements were made. (**B**) Histogram plotting the ISIs for all stimuli delivered in this study.

In all experiments action potentials were individually evoked by injecting a brief current into the soma of B21. Interstimulus intervals (ISIs) were random, and ranged from 0.10 and 3.0 sec (Fig. 2A,B).

To determine whether there was a significant effect of holding potential on PSP induction, stimuli were delivered when the soma was ~10 mV above the normal B21 resting potential (−60 mV), and in a separate run stimuli were delivered when the soma was ~30 mV above resting potential. The contact between B21 and B8 is via its lateral process^19^. Length constant measurements indicate that a somatic depolarization induces a lateral process depolarization that is approximately one half as large^13^. A somatic depolarization of 10 mV is close to the minimal depolarization needed to insure that spikes actively propagate to the lateral process^13^. Consequently we refer to depolarizations of this magnitude as ‘minimal’. Somatic depolarizations of 30 mV are possible because the soma of B21 is inexcitable^20^. They would be expected to induce a depolarization of ~14–15 mV in B21’s lateral process^13^. Peak depolarizations of this magnitude are recorded during motor programs^13^. Central depolarizations larger than 30 mV generally trigger spikes centrally and are no longer subthreshold. Consequently, we refer to +30 mV somatic depolarizations as ‘maximal’.

To determine whether nifedipine had a significant effect on calcium fluorescence or PSP induction, stimuli were delivered when the soma was ~30 mV above resting potential in normal ASW, and after 10 min in 10 μM nifedipine. To evaluate the effect of nifedipine we measured the peak amplitude of the fluorescent signal within a single cell before and after drug application. Because the size of the signal varies from preparation to preparation (presumably due to difference is dye loading, the region imaged, etc) data were normalized before they were pooled. To normalize data they were expressed as a percentage of the smallest signal recorded for that animal. Comparisons were made using a paired t test.

### Data analysis and statistics

In calcium imaging experiments we normalized fluorescence changes by expressing them as a percentage of the mean signal obtained either under control conditions (no nifedipine) or with minimal central depolarization (+10 mV) when the ISI was 1 sec or more (i.e., when facilitation did not occur). Simultaneous measurements of calcium fluorescence and PSP amplitude at different membrane potentials (e.g., Fig. 2A) were obtained from 4 animals. To pool data individual measurements from all animals were combined and sorted by ISI. To generate plots, means and SEMs were computed for every 30 measurements. Ratiometric measurements of alterations in calcium fluorescence resulting from nifedipine application were obtained from 4 animals. Measurements of the effect of nifedipine on PSP amplitude were obtained from 5 animals. To pool PSP data, individual measurements from all animals were combined and sorted by ISI. To generate plots, means and SEMs were computed for every 60 measurements.

Statistical analyses were performed using Prism 8 (Graphpad, San Diego, CA). In all plots error bars indicate SEMs. In all cases, t test comparisons are paired. Results were considered significant when p < 0.05.

## Results

In a previous study we demonstrated that holding potential can modify the dynamics of homosynaptic facilitation at the B21-B8 synapse, i.e., increase the rate at which it occurs^15^. In this previous work B21 was stimulated at pre-selected fixed frequencies. In the present study B21 was stimulated using a protocol in which interstimulus intervals (ISIs) were variable and the ISI bandwidth was 0.10–3.0 sec (Fig. 2A,B). This method made it possible to determine effects of membrane potential on a range of firing frequencies normal for molluscan neurons (not simply preselected values). Further advantages of variable stimulation stemmed from the fact a stimulus delivered at a short ISI was most commonly followed by a stimulus delivered at a longer ISI. Consequently, the average firing frequency was relatively low. This made it possible to monitor the amplitude of individual PSPs in B8 without triggering a postsynaptic response (red arrow in the top trace in Fig. 2A indicates how PSP measurements were made). Since a goal of this study was to determine how changes in background calcium impact facilitation, we simultaneously monitored changes in [Ca^2+^]_i_ in B21 (in addition to PSP induction in B8) (middle trace in Fig. 2A). To measure [Ca^2+^]_i_ we measured changes in calcium fluorescence using a non-ratiometric dye appropriate for monitoring fast changes, i.e., calcium orange or calcium green^21^.

**Figure 3:**
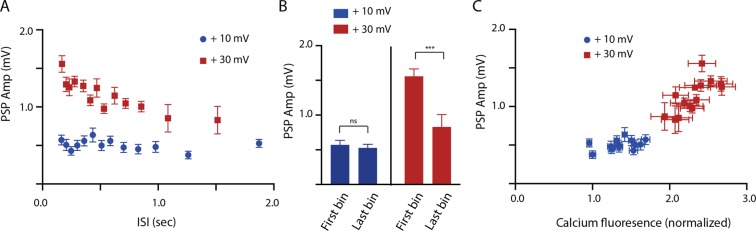
Data obtained using the protocol shown in Fig. 2A. (**A**) Scatter plot of ISI vs. PSP amplitude with minimal central depolarization of B21 (soma 10 mV above resting membrane potential) (blue circles) and with maximal central depolarization (soma 30 mV above resting membrane potential) (red squares). Note that facilitation is only observed with maximal central depolarization. (**B**) Bar graphs plotting the mean PSP amplitude at a short ISI (first bin of pooled data) vs. a long ISI (last bin) with minimal central depolarization (blue) and with maximal central depolarization (red). Note that there was no significant difference with minimal central depolarization, whereas there was a significant difference when B21 was maximally depolarized. (**C**) Scatter plot of normalized calcium fluorescence vs. PSP amplitude with minimal central depolarization of B21 (blue circles) and with maximal central depolarization (red squares). Note that there was no correlation between calcium fluorescence and PSP amplitude with minimal central depolarization. A correlation was however observed with maximal depolarization. In all cases data are plotted as means ± SEMs.

With our stimulation protocol we found that maximal central depolarization was essential for the induction of facilitation (Fig. 3A,B). Thus, when central depolarizations were minimal (~10 mV above resting potential (+10 mV)) there was no correlation between PSP amplitude and ISI (Fig. 3A) (r = −0.30, p = 0.32, 390 measurements from 4 animals). Further, at +10 mV PSPs induced during the first bin of the pooled data (i.e., when the ISI was short) were 0.57 ± 0.05 mV, and PSPs induced during the last bin of the pooled data (i.e., when the ISI was long) were 0.53 ± 0.05 mV (Fig. 3B) (paired t test; t = 0.50, p = 0.62, df = 29, 30 measurements from 4 animals). In contrast, when B21 was maximally depolarized (~30 mV above resting potential (+30 mV)), PSP amplitude and ISI were correlated (Fig. 3A) (r = 0.76, p = 0.0016, 420 measurements from 4 animals). Further, PSPs induced during the first bin were on average 1.56 ± 0.11 mV, whereas PSPs induced during the last bin were on average 1.6 sec were 0.83 ± 0.18 mV (Fig. 3B) (paired t test; t = 5.64, p < 0.0001, df = 29, 30 measurements from 4 animals). Using the random stimulation protocol we therefore identified one condition where facilitation occurs (maximal central depolarization) and one condition where it does not (minimal central depolarization).

To determine whether there was a relationship between the [Ca^2+^]_i_ in B21 and the induction of facilitation we plotted calcium fluorescence vs. PSP amplitude for both the +10 and +30 mV data. When there was no facilitation (+10 mV) calcium fluorescence and PSP amplitude were not correlated (blue circles in Fig. 3C) (r = 0.38, p = 0.20, 390 measurements from 4 animals). In contrast, when there was facilitation (+30 mV) calcium fluorescence and PSP amplitude were correlated (red squares in Fig. 3C) (r = 0.73, p = 0.0029, 420 measurements from 4 animals). These data are consistent with the idea that facilitation does not occur unless there is a certain amount of [Ca^2+^]_i_. Above this value the potentiation observed is related to the size of the calcium signal.

The changes in calcium fluorescence that we monitored in these experiments are all due to widespread increases in the [Ca^2+^]_i_. The highly localized Ca signals that directly trigger transmitter release (Ca_local_) would not be detected with the methods used. Results of previous studies suggest, however, that the measured signal has two components. Namely increases in calcium fluorescence are observed when B21 is depolarized but does not spike^13^. This a graded phenomenon, i.e., increases in fluorescence are determined by the magnitude of the depolarization. Secondly, even when the B21 holding potential is kept constant there are transient spike-induced changes in fluorescence that are presumably a type of Ca_res_^22^. Thus, previous results suggest that the calcium signal measured in experiments such as the one shown in Fig. 2A have a subthreshold depolarization-induced component, and a spike-induced (Ca_res_) component. Other investigators studying facilitation have focused on the role of Ca_res_. The goal of this study was to determine whether the [Ca^2+^]_i_ induced by subthreshold depolarization was making a contribution to the plasticity observed at +30 mV.

**Figure 4:**
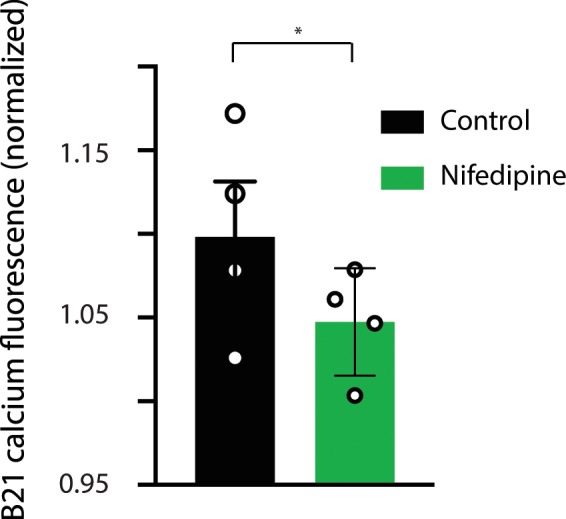
Effect of nifedipine on intracellular calcium. Bar graph plotting normalized calcium fluorescence when B21 was stimulated with random ISIs at +30 mV under control conditions (normal ASW, black bar) and in the presence of 10 μM nifedipine (green bar). In these experiments calcium fluorescence was measured using fura 2 and ratiometric imaging. Note that nifedipine did produce a significant decrease in calcium fluorescence. Data are plotted as means ± SEMs.

If so, we would expect that eliminating (or reducing) it would significantly reduce the size of the summated calcium signal measured when B21 was stimulated at +30 mV. To determine whether this is the case we took advantage of the fact that increases in [Ca^2+^]_i_ induced by subthreshold depolarization are virtually eliminated in 10 μM nifedipine^13^. In contrast, nifedipine has no affect on Ca_res_ induced by spiking. We conducted experiments in which B21 was maximally centrally depolarized and stimulated using the random ISI protocol. Changes in calcium fluorescence were measured before and after nifedipine application. In these experiments changes in calcium fluorescence were imaged ratiometrically. Ratiometric imaging minimizes problems associated with the photobleaching that is likely to occur over the time course of an experiment necessarily prolonged to allow time for drug application and equilibration. We found that 10 μM nifedipine did decrease the amplitude of the calcium signal (Fig. 4) (paired t test; t = 3.27; p = 0.047; df = 3; n = 4). As expected, the effect was relatively small. As discussed above in these experiments the effect of nifedipine was determined on a signal that has both a ‘subthreshold depolarization’ component and a ‘spike-mediated’ component. The spike-mediated component is much larger than the subthreshold depolarization component^22^. Only the subthreshold depolarization component is nifedipine sensitive^22^. Consequently, only a small part of the total signal would be expected to be nifedipine sensitive.

**Figure 5:**
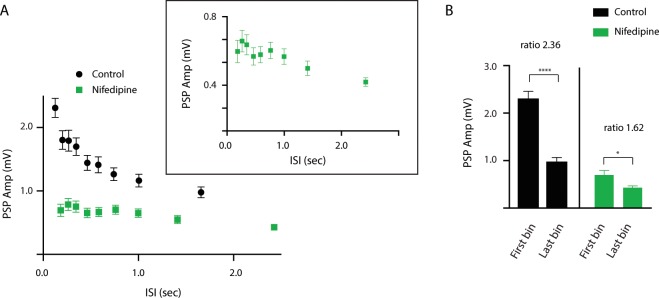
Effect of nifedipine on synaptic transmission. (**A**) Scatter plot of ISI vs. PSP amplitude under control conditions (normal ASW) (black circles) and in the presence of 10 μM nifedipine (green squares). Inset, the data shown in the main part of the figure plotted with a different scaling. Note that facilitation is observed under both conditions, but the facilitation observed in the presence of nifedipine is much less than the facilitation observed under control conditions. (**B**) Bar graphs plotting PSP amplitude at a short ISI (first bin of pooled data) vs. a long ISI (last bin) under control conditions (normal ASW) (black) and in the presence of 10 μM nifedipine (green). Note that the PSP_first bin_/PSP_last bin_ ratio in nifedipine was much less than the PSP_first bin_/PSP_last bin_ ratio under control conditions (i.e., 2.36 vs. 1.62). In all cases data are plotted as means ± SEMs.

In a final set of experiments, we sought to determine whether eliminating the calcium induced by subthreshold depolarization would impact synaptic transmission. B21 was maximally centrally depolarized, and PSPs were monitored in normal ASW and in the presence of 10 μM nifedipine. In normal ASW facilitation occurred (as expected). There was a correlation between PSP amplitude and ISI (r = −0.8685, p = 0.0023, 540 measurements made in 4 animals) (Fig. 5A) and PSPs induced during the first bin (i.e., when the ISIs were short) were on average 2.31 ± 0.15 mV, whereas the PSPs induced during the last bin (i.e., when the ISIs were long) were on average 0.98 ± 0.08 mV (Fig. 5B) (paired t test; t = 7.59; p < 0.0001; df = 59; 60 measurements made from 4 animals). In the presence of nifedipine, facilitation occurred but not to the same extent. There was a correlation between PSP amplitude and ISI (visible in the inset in Fig. 5A) (r = −0.9331; p = 0.0002; 540 measurements made in 4 animals). Further PSPs induced during the first bin were on average 0.70 ± 0.10 mV, whereas PSPs induced during the last bin were on average 0.43 ± 0.04 mV (Fig. 5B) (paired t test; t = 2.51; p = 0.014; df = 59; 60 measurements made from 4 animals). In nifedipine the PSP_first bin_/PSP_last bin_ ratio was, however, much less than the PSP_first bin_/PSP_last bin_ ratio in normal ASW (i.e., 1.62 as opposed to 2.36) (Fig. 5B). These data indicate that the background calcium induced by subthreshold depolarization did in fact contribute to the facilitation observed at the B21/B8 synapse.

## Discussion

Previously we demonstrated that changes in holding potential can modify the dynamics of STE at the B21-B8 synapse^15^. In these experiments B21 was repeatedly stimulated at preselected constant frequencies. For example, 80 stimuli were delivered at 10 Hz at one holding potential, and 80 stimuli were delivered at 10 Hz at a more depolarized potential. At the more depolarized potential facilitation occurred more quickly. In the current study ISIs were variable and stimulation with a short ISI was most commonly followed by stimulation at a longer ISI. In this situation there is less opportunity for facilitation. Interestingly we found that under these conditions the amount of central depolarization can do more than simply modify the dynamics of facilitation. Additionally, it can be essential for determining whether or not facilitation occurs at all.

Although the random ISI protocol we used to activate B21 in these experiments does not mimic a specific physiologically relevant pattern there are undoubtedly situations where high frequency activity in B21 is followed by low frequency spiking. B21 is similar to many other sensory neurons in that it adapts to a maintained stimulus^19^. With adaptation a brief period of high frequency activity is generally followed by low frequency spiking. Our results suggest that when this occurs central depolarization may play a key role in determining whether B21 induced PSPs in B8 facilitate.

### Metaplasticity at the B21-B8 synapse

Modifications of the dynamics and nature of synaptic plasticity have been referred to as ‘metaplasticity’^23^. Although this phenomenon was originally identified as an effect of prior activity on subsequent long term potentiation (LTP) and long term depression (LTD), it has become increasingly apparent that short-term forms of synaptic plasticity can also be plastic^24^. When this is the case plasticity is often altered by neuromodulators. For example, in the lamprey spinal cord substance P and 5-HT impact the amount of synaptic depression and potentiation observed at synapses between identified interneurons and motor neurons^25^. In the stomatogastric pyloric circuit the graded component of the LP to PD synapse depresses under control conditions, but is enhanced and can show facilitation in the presence of the neuropeptide proctolin^26^. The metaplasticity described here differs in that it does not require a change in modulatory input.

In B21, metaplasticity is observed when there is a centrally induced change in membrane potential. Under physiological conditions changes in the B21 membrane potential occur when the feeding neural network is activated^27–29^. For example, B21 is centrally depolarized via electrical and fast synaptic input from pattern generating interneurons^16,27^. This occurs during one of the two phases of ingestive motor programs. Previous work established that these central depolarizations gate-in afferent activity at the behaviorally appropriate time^13,29^. Activation of the B21/B8 circuitry potentially results in radula closing in response to food contact. During ingestive behavior it is important that this occurs during radula retraction so that food will be pulled into the buccal cavity. If the radula closes during protraction food will be pushed out.

Previous studies have shown that phase-dependent control of B21-B8 transmission is in part mediated by the regulation of spike propagation in B21^20,29,30^. When B21 is peripherally activated at its resting membrane potential, spikes do not actively propagate to the process that contacts B8 (the lateral process). In contrast, when B21 is centrally depolarized the spike propagation failure is relieved. Interestingly, however, previous work has also shown that central depolarization does more than modify spike propagation. Additionally, there are important effects on the efficacy of synaptic transmission. Thus, when central depolarizations are just barely enough to alter spike propagation in B21 PSPs in B8 are so small they are difficult to detect^13^. Further central depolarization is therefore necessary if effective sensorimotor transmission is to occur. Central depolarization increases PSP size when B21 fires at a very low frequency and facilitation does not occur, but unfacilitated PSPs are still relatively small and do not effectively activate B8^13^. Facilitation at the B21/B8 synapse is therefore likely to be an important part of the gating in of afferent input^15^. To summarize, work in other systems has established that metaplasticity can occur when there is a state change and an alteration in modulatory input. The results of this study taken together with previous work demonstrate that metaplasticity can also impact synaptic transmission in a more dynamic (e.g., phase dependent) manner.

### Background calcium and STE at the B21/B8 synapse

A previous study demonstrated that changes in its holding potential alter the amount of background calcium in B21^13^. Namely, when B21 is depolarized the [Ca^2+^]_i_ is increased. We hypothesized that these increases in [Ca^2+^]_i_ were at least partially responsible for membrane potential-induced alterations in STE. To determine whether this is the case we compared the STE observed under control conditions (normal ASW) to the STE observed in the presence of nifedipine. Nifedipine blocks a calcium current induced in B21 at relatively low voltages^31^ and either reduces or eliminates increases in background calcium resulting from subthreshold changes in membrane potential^13^. We found that in the presence of nifedipine the facilitation that we observed was greatly reduced. This indicates that increases in background calcium are in fact at least partially responsible for membrane potential-induced alterations in STE at the B21/B8 synapse.

In other systems investigators studying mechanisms responsible for STE have focused on the importance of the calcium that remains in a neuron after an action potential. Thus, when a neuron spikes, high voltage activated (HVA) calcium channels near the active zone open resulting in a large calcium signal that does not last long and drops off significantly with distance from the opened channels. This highly localized increase in [Ca^2+^]_i_ (Ca_local_) generally triggers synchronous transmitter release. Although the HVA calcium channels necessary for release do not remain open long, calcium diffuses and equilibrates with calcium-binding proteins. Consequently, a much smaller persistent calcium signal (Ca_res_) is also generated. It has been suggested that Ca_res_ induces facilitation, e.g., by binding to a high affinity sensor such as synaptotagmin 7 and increasing the release probability (*p*)^32^. Our results suggest that at least in some systems facilitation can also be impacted by calcium entering the neuron through other channels (e.g., relatively LVA activated channels opened by subthreshold depolarization). When this occurs there is a relatively dynamic modification of synaptic transmission.

## Data Availability

The datasets generated during and/or analyzed during the current study are available from the corresponding author on reasonable request.

## References

[1] Byrne JH, Kandel ER (1996). Presynaptic facilitation revisited: state and time dependence. J. Neurosci..

[2] Zucker RS, Regehr WG (2002). Short-term synaptic plasticity. Annu. Rev. Physiol..

[3] Awatramani GB, Price GD, Trussell LO (2005). Modulation of transmitter release by presynaptic resting potential and background calcium levels. Neuron.

[4] Alle H, Geiger JR (2006). Combined analog and action potential coding in hippocampal mossy fibers. Science.

[5] Alle H, Geiger JR (2008). Analog signalling in mammalian cortical axons. Curr. Opin. Neurobiol..

[6] Clark B, Hausser M (2006). Neural coding: hybrid analog and digital signalling in axons. Curr. Biol..

[7] Marder E (2006). Neurobiology: extending influence. Nature.

[8] Shu Y, Hasenstaub A, Duque A, Yu Y, McCormick DA (2006). Modulation of intracortical synaptic potentials by presynaptic somatic membrane potential. Nature.

[9] Shimahara T, Tauc L (1975). Multiple interneuronal afferents to the giant cells in Aplysia. J. Physiol..

[10] Nicholls J, Wallace BG (1978). Modulation of transmission at an inhibitory synapse in the central nervous system of the leech. J. Physiol..

[11] Ivanov AI, Calabrese RL (2003). Modulation of spike-mediated synaptic transmission by presynaptic background Ca2+ in leech heart interneurons. J. Neurosci..

[12] Ivanov AI, Calabrese RL (2006). Spike-mediated and graded inhibitory synaptic transmission between leech interneurons: evidence for shared release sites. J. Neurophysiol..

[13] Ludwar B, Evans CG, Jing J, Cropper EC (2009). Two distinct mechanisms mediate potentiating effects of depolarization on synaptic transmission. J. Neurophysiol..

[14] Christie JM, Chiu DN, Jahr CE (2011). Ca(2+)-dependent enhancement of release by subthreshold somatic depolarization. Nat. Neurosci..

[15] Evans CG, Ludwar B, Askanas J, Cropper EC (2011). Effect of holding potential on the dynamics of homosynaptic facilitation. J. Neurosci..

[16] Rosen SC, Miller MW, Evans CG, Cropper EC, Kupfermann I (2000). Diverse synaptic connections between peptidergic radula mechanoafferent neurons and neurons in the feeding system of Aplysia. J. Neurophysiol..

[17] Klein AN, Weiss KR, Cropper EC (2000). Glutamate is the fast excitatory neurotransmitter of small cardioactive peptide-containing Aplysia radula mechanoafferent neuron B21. Neurosci. Lett..

[18] Ludwar, B., Evans, C. G. & Cropper, E. C. Monitoring changes in the intracellular calcium concentration and synaptic efficacy in the mollusc Aplysia. *J. Vis. Exp.*, e3907, 10.3791/3907 (2012). 10.3791/3907 PMC3476414 22824826

[19] Borovikov D, Evans CG, Jing J, Rosen SC, Cropper EC (2000). A proprioceptive role for an exteroceptive mechanoafferent neuron in Aplysia. J. Neurosci..

[20] Evans CG, Ludwar B, Cropper EC (2007). Mechanoafferent neuron with an inexcitable somatic region: consequences for the regulation of spike propagation and afferent transmission. J. Neurophysiol..

[21] Eberhard M, Erne P (1991). Calcium-Binding to Fluorescent Calcium Indicators - Calcium Green, Calcium Orange and Calcium Crimson. Biochem. Bioph. Res. Co..

[22] Ludwar BC, Evans CG, Cambi M, Cropper EC (2017). Activity-dependent increases in [Ca(2+)]i contribute to digital-analog plasticity at a molluscan synapse. J. Neurophysiol..

[23] Abraham WC, Bear MF (1996). Metaplasticity: the plasticity of synaptic plasticity. Trends. Neurosci..

[24] Nadim F, Manor Y (2000). The role of short-term synaptic dynamics in motor control. Curr. Opin. Neurobiol..

[25] Parker D, Grillner S (1999). Activity-dependent metaplasticity of inhibitory and excitatory synaptic transmission in the lamprey spinal cord locomotor network. J. Neurosci..

[26] Zhao S, Sheibanie AF, Oh M, Rabbah P, Nadim F (2011). Peptide neuromodulation of synaptic dynamics in an oscillatory network. J. Neurosci..

[27] Rosen SC, Miller MW, Cropper EC, Kupfermann I (2000). Outputs of radula mechanoafferent neurons in Aplysia are modulated by motor neurons, interneurons, and sensory neurons. J. Neurophysiol..

[28] Shetreat-Klein AN (2004). & Cropper, E. C. Afferent-induced changes in rhythmic motor programs in the feeding circuitry of aplysia. J. Neurophysiol..

[29] Evans CG, Jing J, Rosen SC, Cropper EC (2003). Regulation of spike initiation and propagation in an Aplysia sensory neuron: gating-in via central depolarization. J. Neurosci..

[30] Evans CG, Kang T, Cropper EC (2008). Selective spike propagation in the central processes of an invertebrate neuron. J. Neurophysiol..

[31] Svensson E, Evans CG, Cropper EC (2016). Repetition priming-induced changes in sensorimotor transmission. J. Neurophysiol..

[32] Jackman SL, Turecek J, Belinsky JE, Regehr WG (2016). The calcium sensor synaptotagmin 7 is required for synaptic facilitation. Nature.

